# Establishment of Tissue Culture and Genetic Transformation Systems Using Mature Embryos of Bread Wheat

**DOI:** 10.3390/plants15182766

**Published:** 2026-09-10

**Authors:** Yiyang He, Yingrun Wang, Jia Shi, Songyu Pang, Wenyang Li, Chuanzhi Wang, Lantian Ren, Zhaoshi Xu, Dong Wang, Jiacheng Zheng

**Affiliations:** 1 College of Agriculture, Anhui Science and Technology University/Bio-Breeding Laboratory of Anhui Province, Chuzhou 233100, China; 2 College of Biology and Food Engineering, Suzhou University, Suzhou 234000, China; 3 Anhui Engineering Research Center for Smart Crop Planting and Processing Technology, Chuzhou 233100, China; 4 Institute of Crop Science, Chinese Academy of Agricultural Sciences (CAAS)/National Key Facility for Crop GeneResources and Genetic Improvement, Key Laboratory of Biology and Genetic Improvement of Triticeae Crops, Ministry of Agriculture, Beijing 100081, China; 5 College of Agriculture, Northwest A&F University/National Key Laboratory of Crop Stress Resistance and Efficient Production, Xianyang 712100, China

**Keywords:** wheat, mature embryos, genetic transformation, regeneration system, callus induction

## Abstract

Genetic transformation is a core technology for wheat molecular breeding. Transformation efficiency and genotype compatibility determine the pace of targeted genetic improvement. Mature embryos are readily accessible but are associated with low transformation efficiency and strong genotype dependence. Using mature wheat embryos as explants, we screened eight wheat varieties representing three ecological types to develop genotype-adapted tissue culture systems. Treatments A5 (4 mg/L 2,4-D + 2 mg/L Dicamba), A7 (4 mg/L 2,4-D + 6 mg/L Dicamba), and A8 (4 mg/L 2,4-D + 8 mg/L Dicamba) were optimal for callus induction in winter, semi-winter, and spring wheat, respectively. WF3 (a 1/2 MS-based medium) was broadly applicable to callus differentiation, whereas IBA produced the highest rooting efficiency in regenerated plantlets. The optimal transformation conditions comprised ultrasound treatment (2.5 min) followed by vacuum infiltration (5 min), supplementation of the infection solution with PEG_6000_ (250 mg/L) and Tween20 (0.01%), and an OD_600_ of 0.6–0.8. Transformation efficiencies using mature embryos of the semi-winter wheat varieties YX288 and ZM578 were 2.5% and 1.8%, respectively. Using this transformation platform, the dwarfing genes *Rht12* in ZM578 and *Rht8* in YX288 were targeted by CRISPR/Cas9-mediated editing. Multiple single-base substitutions at the target sites were detected in ZM578 *rht12* mutants, which exhibited significantly reduced height, increased tiller number, and a compact plant architecture. However, no successfully edited *rht8* lines were obtained in YX288. These findings indicate that this study established a reliable mature-embryo transformation system for wheat and successfully validated gene editing in the semi-winter variety ZM578. The system provides a technical platform for the functional analysis of key genes and molecular breeding in bread wheat.

## 1. Introduction

An efficient and stable genetic transformation system is essential for molecular breeding and functional gene studies in bread wheat [1,2]. Currently, wheat genetic transformation relies predominantly on immature embryos as explants [3]. However, this approach is severely constrained by the growing season and specific developmental stages of the donor plants [4]. It also exhibits strong genotype dependence, leading to substantial variation in transformation efficiency and experimental reproducibility [2,5]. These limitations impede the functional validation of key genes and restrict the large-scale application of molecular breeding technologies [1,2]. Mature embryos, which are fundamental structures in seed germination, contain a complete apical meristem and organ primordia and theoretically possess strong regenerative potential [6,7]. Compared with immature embryos, mature embryos offer distinct advantages, including easy collection, independence from the growing season, and convenient storage [6,8]. Recent callus-culture studies have identified optimization of in vitro regeneration from mature embryos as critical to overcoming the bottleneck in wheat transformation. Replacing 2,4-D with picloram or dicamba, or combining these auxins, effectively induces a high-quality, compact callus with typical embryogenic characteristics in durum wheat and several common wheat cultivars, thereby substantially improving green-shoot regeneration [9].

Although hormone-regulation strategies for mature-embryo regeneration in wheat have advanced, several technical bottlenecks remain. Pronounced genotype dependence is the primary limitation because protocols optimized for model varieties are difficult to adapt to major commercial cultivars [6]. The biological characteristics of mature embryos further complicate their use. Their dense cell walls and low metabolic activity form physical and physiological barriers to *Agrobacterium* infection [3]. They may also exhibit an exaggerated oxidative response during infection, triggering a reactive oxygen species (ROS) burst and activating innate immune defenses in wheat explants [10,11]. This combined stress response causes browning and necrosis of recipient tissues, resulting in low and unstable transformation efficiency [6,7,8]. In addition, the prolonged in vitro culture required for mature embryos can induce somaclonal variation. The extent of this variation may differ markedly with chromosome ploidy and genotype, further increasing disparities in regeneration capacity among the materials [10,11,12].

To overcome the low transformation efficiency and strong genotype dependence of wheat mature-embryo transformation, this study used mature embryos from eight major wheat varieties. We systematically screened basal medium components and hormone ratios to identify optimal formulations for winter, semi-winter, and spring wheat varieties. Distinct callus-induction protocols were developed according to the biological characteristics of the three ecological types. Key conditions for differentiation and rooting were also optimized to establish a standardized tissue-culture regeneration system. On this basis, *Agrobacterium* carrying the *GUS* reporter gene was used to transform varieties from all three ecological types. Core transformation parameters, including bacterial suspension concentration, coculture conditions, and medium composition, were optimized. The utility of the system was then evaluated by CRISPR/Cas9-mediated editing of the gibberellin-sensitive dwarfing genes *Rht8* and *Rht12.* The aim was to generate *rht8* and *rht12* mutant germplasm with a compact, high-tillering architecture and no adverse effects on yield. Overall, this study sought to establish an efficient, stable, and broadly applicable genetic transformation system based on mature embryos of bread wheat, thereby providing technical support for gene-function validation and molecular breeding.

## 2. Results

### 2.1. Establishment of Tissue Culture Systems for Winter, Semi-Winter, and Spring Wheat Varieties

#### 2.1.1. Combined Effects of 2,4-D and Dicamba on Callus Induction

**Figure 1:**
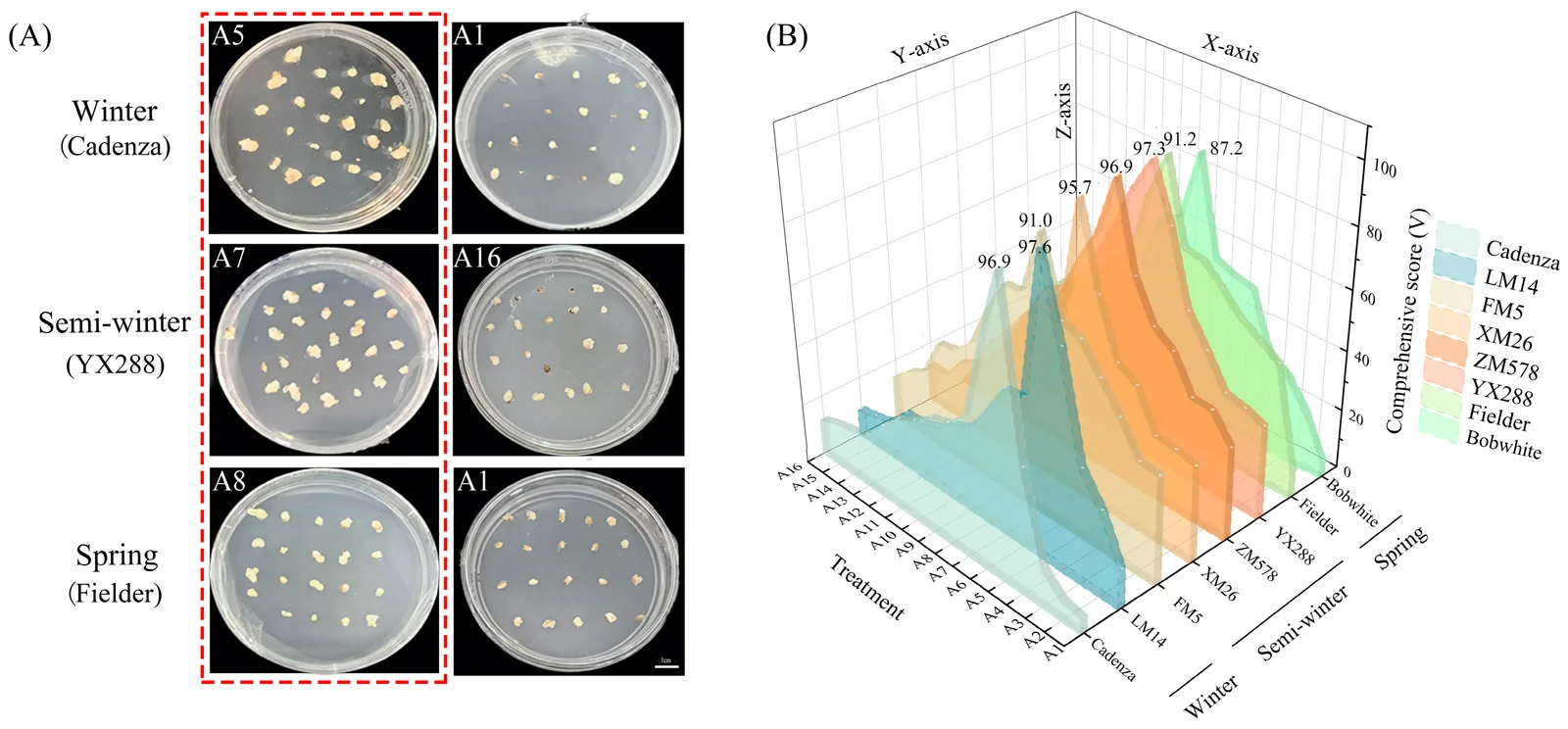
Selection of induction media for winter, semi-winter, and spring varieties. (**A**): The calli induction examples of winter, semi-winter, and spring varieties on the worst and best media, the red box represents the best. (**B**): Comprehensive score of eight varieties under different concentration combinations of 2,4-D and Dicamba. Comprehensive score (V) = (Induction rate score × 30%) + (Dry weight score × 50%) + (Fresh weight score × Dynamic weight). The dynamic weight was adjusted according to the calli moisture content; if the moisture content was higher than 90%, only 10% of the weight was retained, otherwise, 20% was retained.

To develop a broadly applicable callus-culture system, we evaluated the combined effects of 2,4-D and dicamba in eight selected varieties (Figure 1). Wheat varieties from different ecological types exhibited distinct genotype-specific responses to the hormone ratios. The winter varieties Cadenza and LM14 performed the best under treatment A5 (4 mg/L 2,4-D + 2 mg/L Dicamba). This result indicated that these genotypes required a higher concentration of the auxin analog dicamba to initiate efficient dedifferentiation. Treatment A8 (4 mg/L 2,4-D + 8 mg/L Dicamba) was optimal for the spring wheat varieties Bobwhite and Fielder, indicating that an even higher dicamba concentration was required to induce callus formation in the spring genotypes.

#### 2.1.2. Effects of Different Differentiation Media on Callus Differentiation

**Figure 2:**
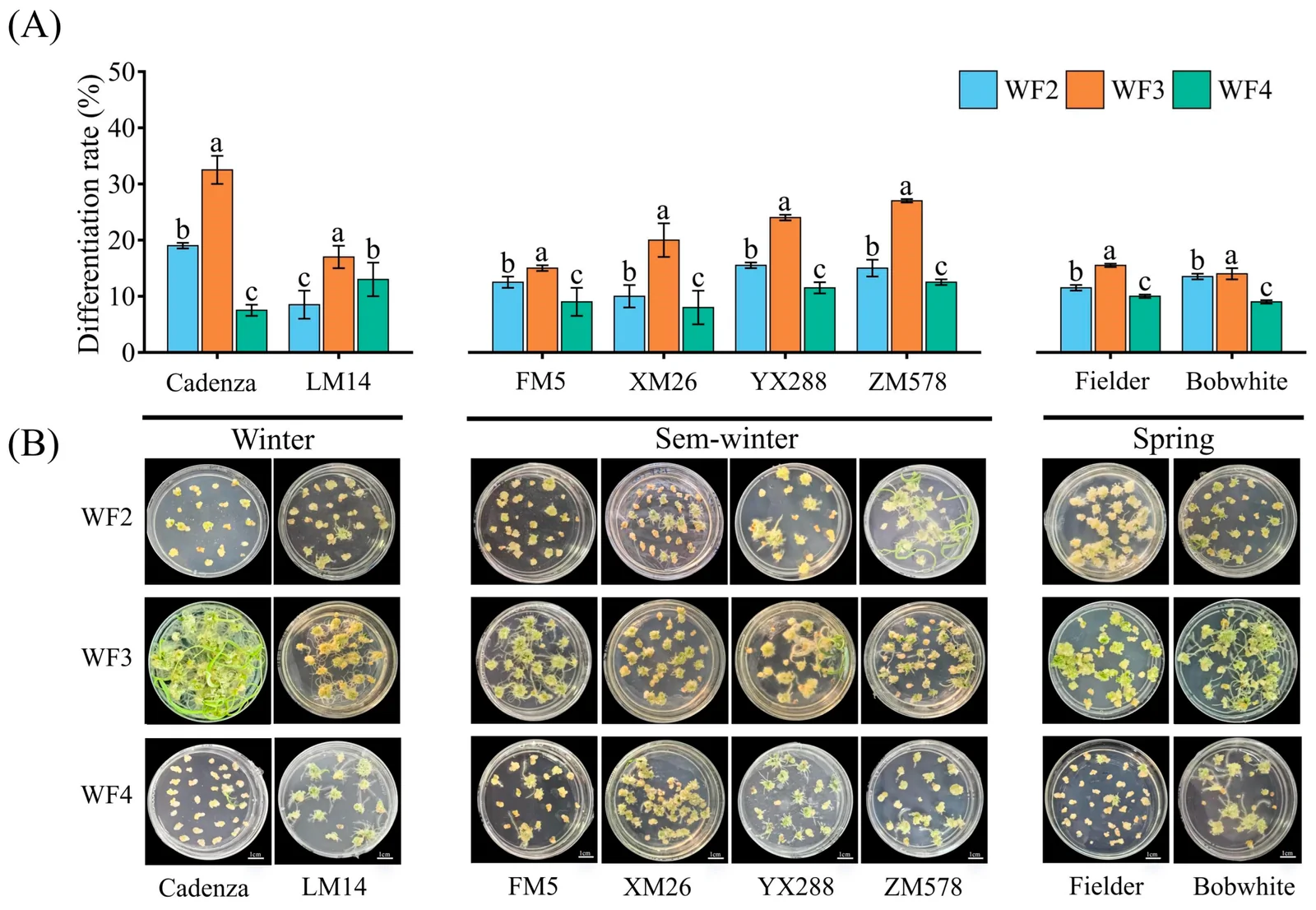
Differentiation effects of three media on mature embryos of winter, semi-winter and spring wheat. (**A**): The differentiation rates of diverse varieties with different media; (**B**): The calli differentiation status of different varieties. Lowercase letters represent significant differences for the same variety under different media *(p* < 0.05, *n* = 15).

Three differentiation media (WF2, WF3, and WF4) were tested to evaluate the differentiation status of eight wheat varieties. All eight varieties achieved the highest differentiation rate on medium WF3 (1/2 MS), which was significantly higher than those on WF2 (full MS) and WF4 (1/4 MS) (Figure 2). These findings indicated that WF3 was broadly applicable to varieties from most ecological types. WF3 contains half the inorganic salt concentration of full-strength MS medium, resulting in osmotic pressure and ionic strength that may better support embryogenic calli during shoot regeneration. These conditions effectively promoted the differentiation of embryogenic cell clusters and the formation of green shoots. For WF3, Cadenza, XM26, YX288, and ZM578 exhibited relatively high differentiation rates.

#### 2.1.3. Effects of Different Rooting Media on Root Growth in Regenerated Seedlings

With IBA treatment, total root length, root surface area, root volume, number of root tips, and number of root branches in the eight varieties were significantly greater than with the other two treatments (Table 1). The IBA + NAA treatment ranked second for total root length, root surface area, average root diameter, and number of root tips. With NAA treatment, average root diameter was greatest, followed by root volume and the number of root branches, whereas the other root traits showed weaker responses. These results indicated that the IBA medium was optimal for root development in regenerated seedlings.

### 2.2. Establishment of Genetic Transformation Systems for Winter, Semi-Winter, and Spring Wheat Varieties

#### 2.2.1. Effect of AgNO_3_ Supplementation in the Coculture Medium on Transformation Efficiency

**Figure 3:**
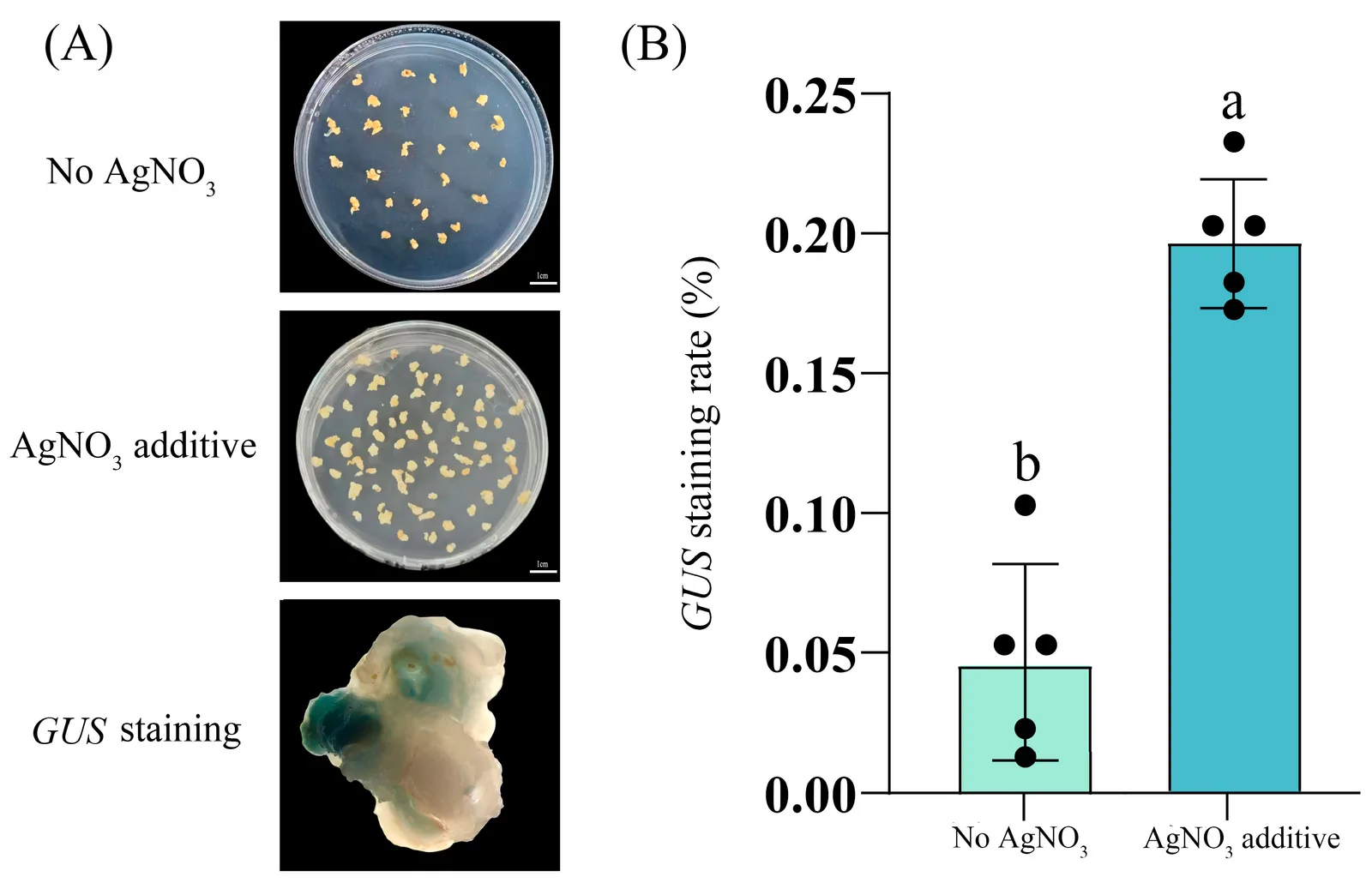
Effects of AgNO_3_ addition to *Agrobacterium* on Cadenza transformation efficiency. (**A**): Effects of AgNO_3_ additive on calli recovery. (**B**): Effects of AgNO_3_ on *GUS* staining rate. Lowercase letters represent significant differences (*p* < 0.05, *n* = 15).

Using the reported model variety Cadenza, we analyzed the effects of antioxidant AgNO_3_ in the coculture medium on wheat transformation efficiency (Figure 3). Compared with the control without AgNO_3_, AgNO_3_ supplementation accelerated callus recovery after infection, significantly reduced browning, and improved callus quality and growth. These findings indicated that AgNO_3_ alleviated oxidative stress in calli during *Agrobacterium* infection and coculture, thereby providing more favorable conditions for postinfection survival and proliferation. Calli in both the control and AgNO_3_-treated groups stained positive for *GUS*. The *GUS* staining rate was significantly higher in the AgNO_3_-treated group than in the control group, indicating that AgNO_3_ supplementation was associated with greater transient *GUS* expression. Nevertheless, the *GUS* staining rate remained low and required further optimization.

#### 2.2.2. Ultrasound- and Vacuum-Infiltration-Assisted Agrobacterium Infection of Calli

**Figure 4:**
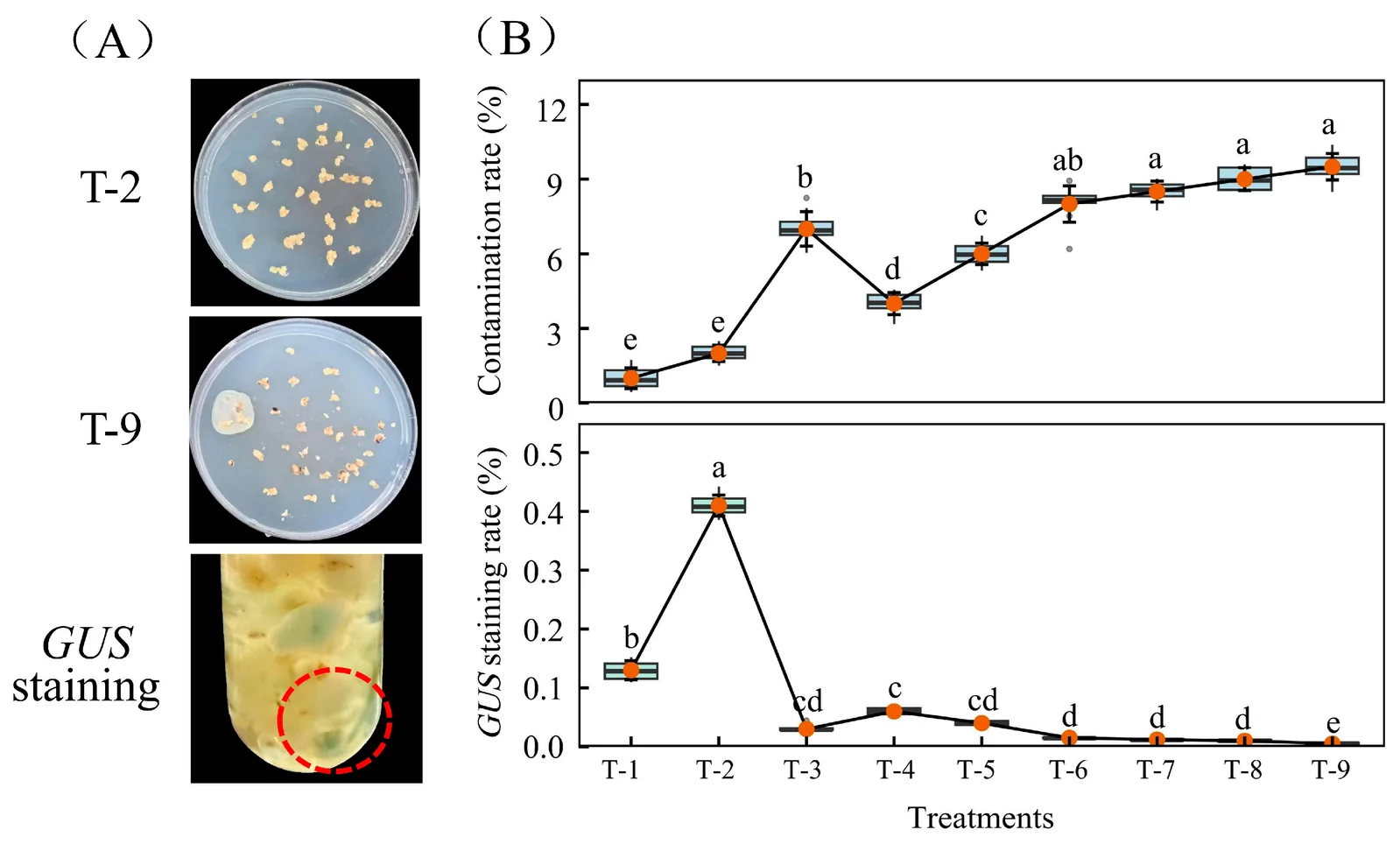
Assisted effects of ultrasound and vacuum infiltration on the *Agrobacterium* infection of calli. (**A**) The growth status and *GUS* staining of calli under the best (T-2) and worst (T-9) treatments. The red dotted circles indicate the *GUS*-stained positive calli. (**B**) *GUS* staining rate and contamination rate. Lowercase letters indicate significant differences among different treatments for the same indicator at the 0.05 level (*p* < 0.05, *n* = 15).

To improve the low efficiency of *Agrobacterium*-mediated transformation, we evaluated two physical assistance methods: ultrasound and vacuum infiltration (Figure 4). In the model variety Cadenza, treatment T-2 (2.5 min ultrasound + 5 min vacuum infiltration) produced the best overall results, followed by T-1 (2.5 min ultrasound + 2.5 min vacuum infiltration); the treatments differed significantly in *GUS* staining and contamination rates. Treatment T-9 (15 min ultrasound + 15 min vacuum infiltration) resulted in the most severe callus necrosis and contamination. *GUS* staining confirmed the successful transformation of Cadenza calli under T-2, with a staining rate of 0.41%, twice that obtained with AgNO_3_ supplementation alone. These findings indicated that T-2 improved T-DNA transfer efficiency without causing substantial physical damage to the calli. In contrast, prolonged ultrasound exposure, as in T-8 and T-9, significantly increased callus contamination and reduced the *GUS* staining rate, suggesting that excessive physical stress caused mechanical damage and created favorable conditions for microbial contamination.

#### 2.2.3. Screening of Different Wheat Genotypes Based on the Primary Transformation System

**Figure 5:**
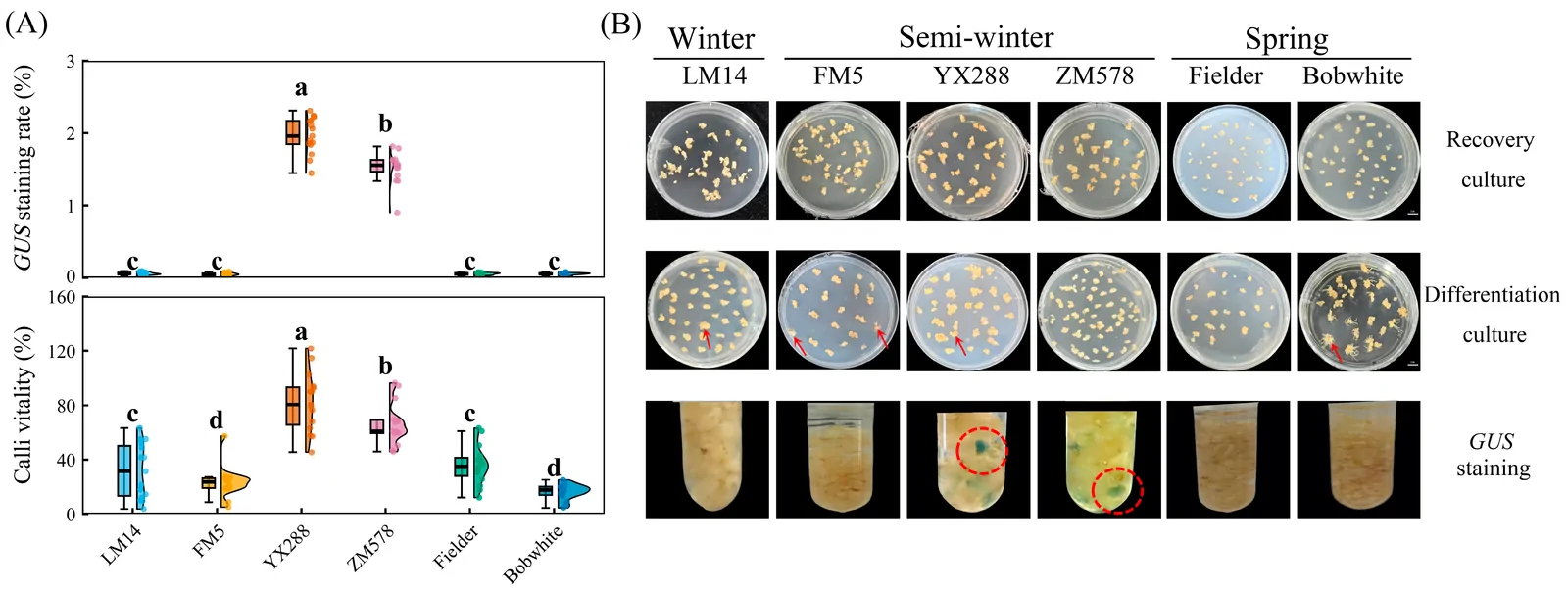
Screening of different wheat genotypes based on primary transformation system. (**A**): The calli vitality and *GUS* staining rate. (**B**): Calli growth and *GUS* staining after *Agrobacterium* infection. The red arrows indicate the differentiated calli without shoots, and the red circles indicate the *GUS*-stained positive calli. Lowercase letters represent significant differences among different varieties *(p* < 0.05, *n* = 15).

Based on the optimized transformation parameters (coculture with 4 mg/L AgNO_3_ and physical assistance with T-2), six varieties (LM14, FM5, YX288, ZM578, Fielder, and Bobwhite), representing the three ecological types, were selected for transformation (Figure 5). Callus vitality was significantly higher in the semi-winter varieties YX288 and ZM578 than in the other varieties, and their calli showed the most vigorous growth. The calli from both varieties were positive for *GUS* staining; their *GUS* staining rates during differentiation were 2.1% and 1.5%, respectively. The calli proliferated vigorously and showed little browning. However, transformed calli of both varieties failed to regenerate seedlings, possibly because of stresses associated with *Agrobacterium* infection and antibiotic selection. YX288 and ZM578 were therefore selected for further optimization of the infection parameters.

#### 2.2.4. Effects of Additives on the Recovery Capacity of Calli from Selected Wheat Genotypes

**Figure 6:**
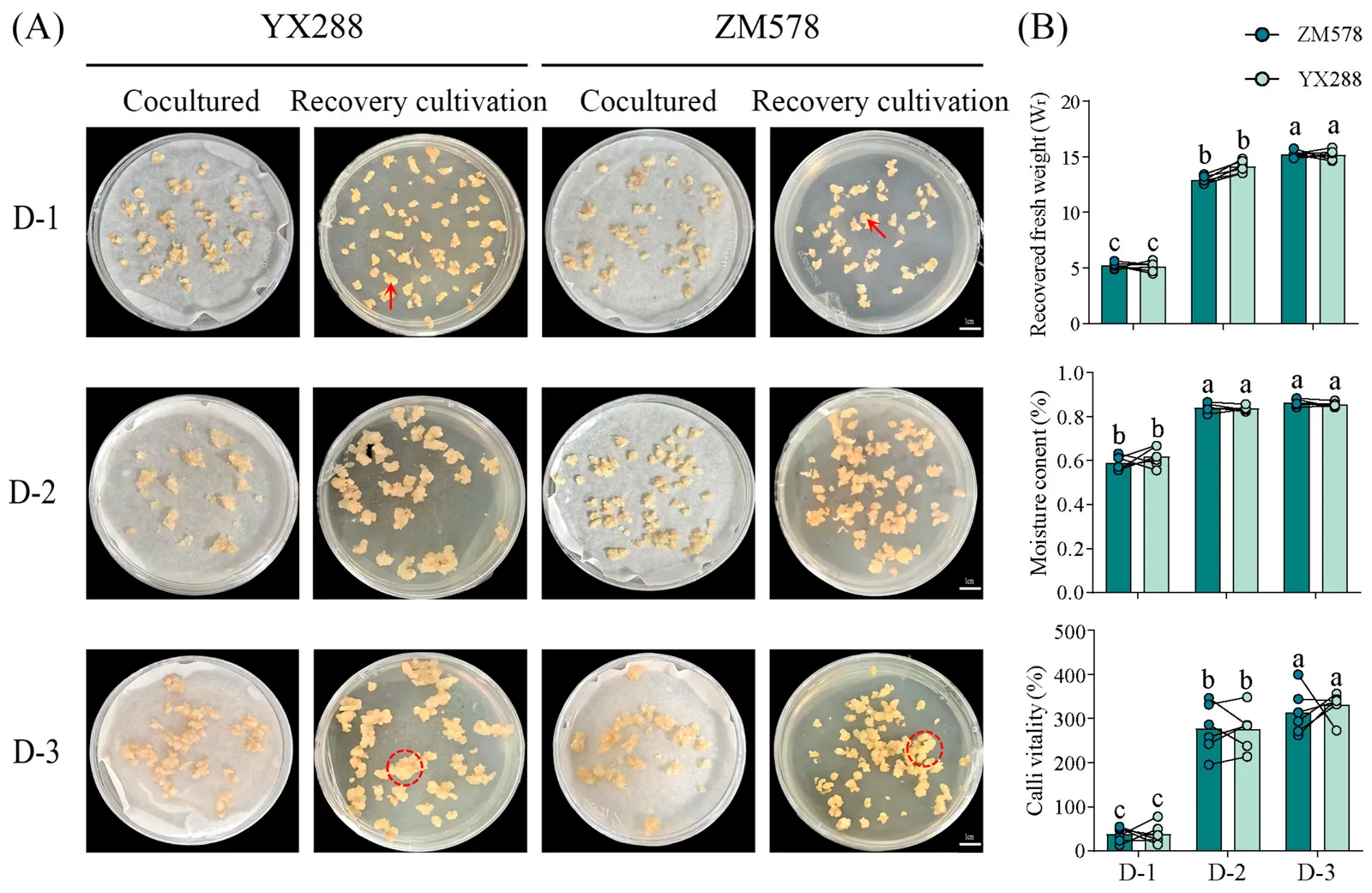
Effects of additives in the infection solution on the recovery capacity of calli. (**A**): Growth status of calli after recovery culture. The red arrows indicate the calli with poor growth status, whereas the red circles indicate calli with good growth status. (**B**): Fresh weight, calli vitality, and moisture content after recovery culture. The infection solution was mixed with different additives, including D-1 (0.02% Silwet L-77), D-2 (250 mg/L PEG_6000_ + 0.02% Silwet L-77), and D-3 (250 mg/L PEG_6000_ + 0.01% Tween20). Lowercase letters represent significant differences among three treatments for the same variety (*p* < 0.05, *n* = 15).

Using the optimized transformation parameters, different additives were incorporated into the *Agrobacterium* infection solution to improve callus vitality. During coculture, calli showed the best recovery under D-3, with a bright-yellow color and high physiological activity. D-2 ranked second, and some calli showed signs of recovery (Figure 6). Under D-1, callus recovery was slow; most calli were pale, showed localized browning, and exhibited nearly arrested growth. In YX288 and ZM578, fresh weight (W_r_) and callus vitality were highest under D-3 and differed significantly from those under D-1 and D-2. In addition, the moisture content of calli treated with D-2 and D-3 was below 90%, indicating a relatively balanced relationship between water status and biomass accumulation. These findings indicated that D-3 (Tween20 + PEG_6000_) effectively alleviated cellular damage following *Agrobacterium* infection.

#### 2.2.5. Effects of Agrobacterium Concentration on Callus Differentiation and the GUS Staining Rate

**Figure 7:**
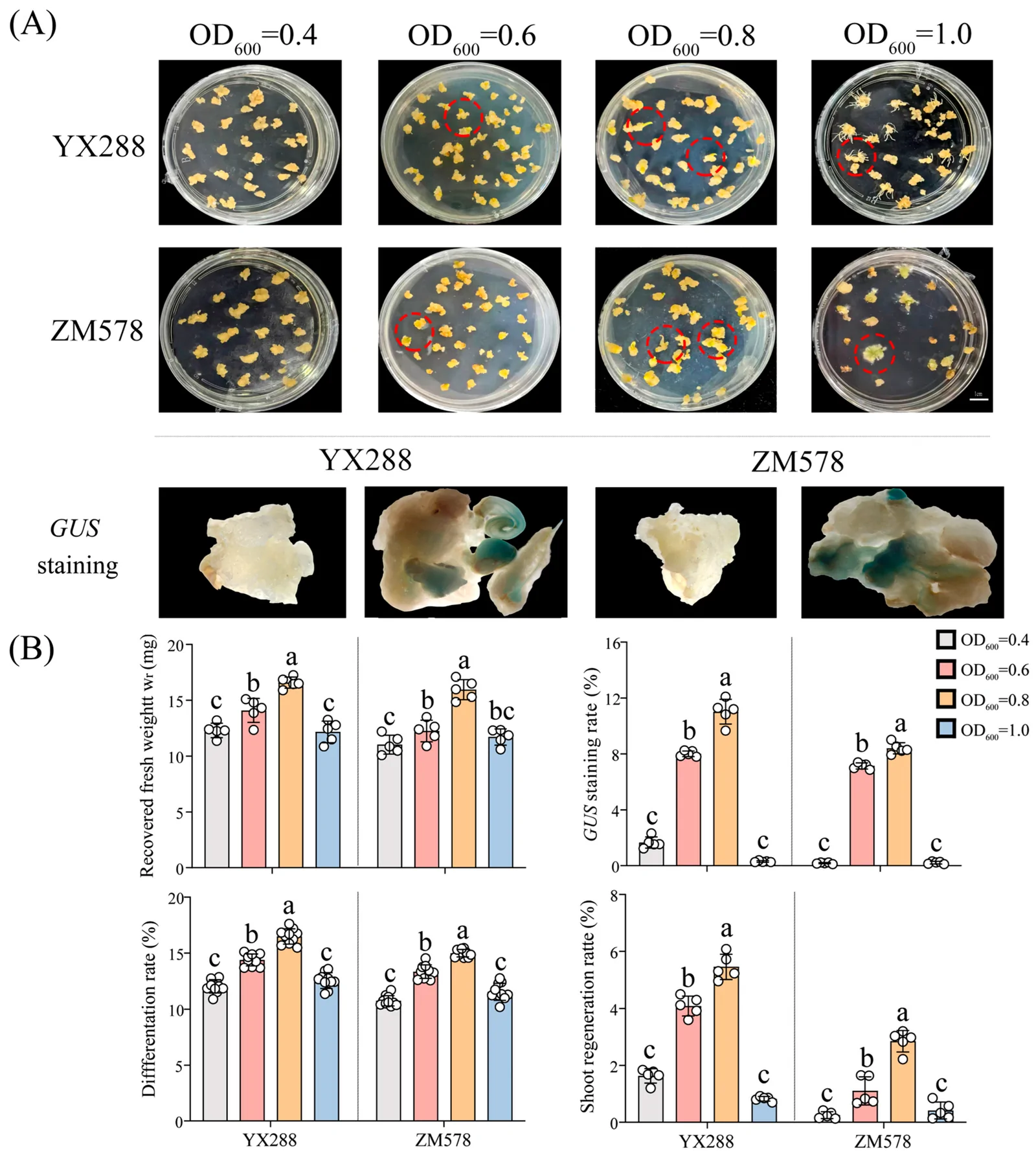
Effects of *Agrobacterium* concentrations on the differentiation of calli and the *GUS* staining rate. (**A**): Growth status and *GUS* staining of calli after differentiation for 20 days. The red circles indicate the callus growth status. (**B**): *GUS* staining rate and regeneration-related indicators of calli. Lowercase letters represent the significant differences under different *Agrobacterium* concentrations for the same variety in each indicator (*p* < 0.05, *n* = 15).

After 20 days of callus differentiation, YX288 and ZM578 calli showed clear green-bud differentiation at OD_600_ = 0.6 and OD_600_ = 0.8, whereas no differentiation was observed at OD_600_ = 0.4 (Figure 7). At OD_600_ = 1.0, numerous adventitious roots formed in the calli, whereas green-bud differentiation was inhibited. At OD_600_ = 0.8, the calli of YX288 and ZM578 had the greatest fresh weight (W_r_) after recovery and the highest *GUS* staining rates, which were 10.7% and 7.4%, respectively; these values differed significantly from those under the other treatments. After 30 days of differentiation at OD_600_ = 0.8, the differentiation rates of YX288 and ZM578 were 16.49% and 14.99%, respectively, and their shoot regeneration rates were 5.46% and 2.85%, respectively. Both measures were significantly higher than those under the other treatments.

#### 2.2.6. Detection of Transgenic Seedlings

**Figure 8:**
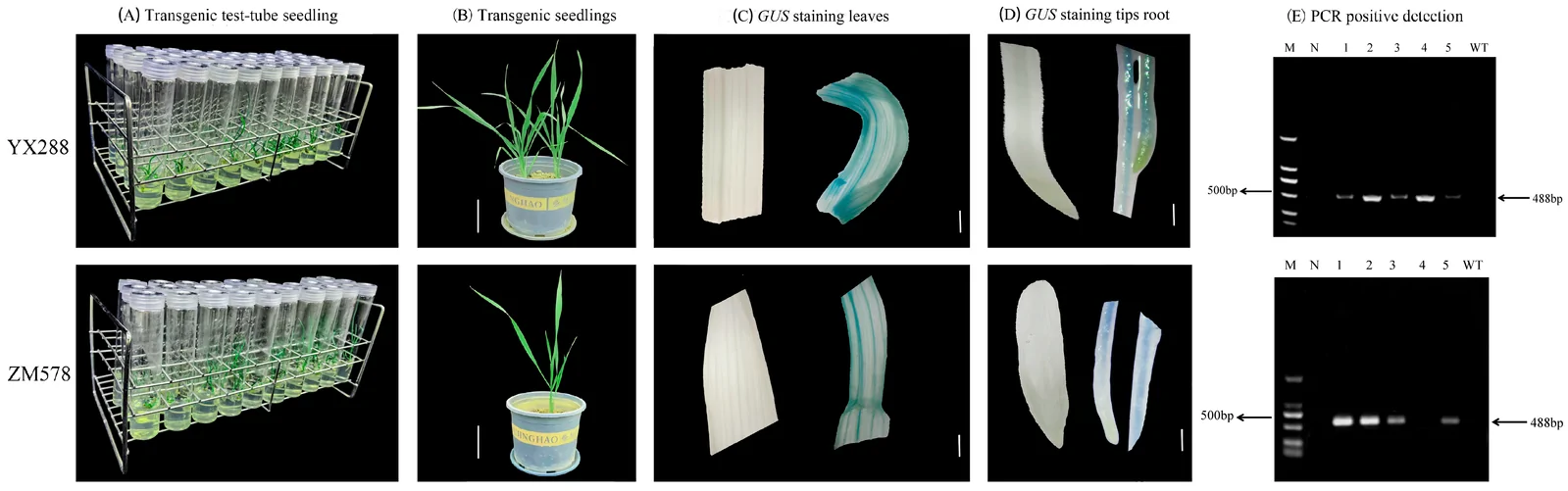
PCR positive detection and *GUS* tissue staining of transgenic seedlings. (**A**): Transgenic test-tube seedlings. (**B**): Transgenic seedlings after 100 days of growth, a scale of 10 cm. (**C**,**D**): *GUS* staining with leaves and root tips, respectively, at a scale of 1 mm. (**E**): PCR positive detection, M is the DL2000 Marker (Protein, Beijing, China), N is the negative control, lanes 1–6 represent the transgenic seedlings detection, and WT represents the wild-type lines.

After 100 days of transgenic seedling culture, leaves and root tips were subjected to *GUS* staining. The in vitro plantlets were transplanted into nutrient pots and cultivated until the fourth leaf had emerged; each plant was then labeled for PCR analysis (Figure 8). The leaf veins and mesophyll cells of transgenic YX288 and ZM578 plants stained blue, whereas those of wild-type (WT) control plants did not. Similarly, the root tips of transgenic plants stained blue, whereas those of WT plants did not, indicating stable *GUS* expression in the transgenic plants. PCR amplification produced a clear and specific *GUS* target band of 488 bp in the transgenic plants, further confirming the presence of the exogenous gene. Overall, seedling regeneration rates were 3.6% and 2.5% for YX288 and ZM578, respectively; transformation efficiencies were 2.5% and 1.8%, respectively; and PCR-positive rates were 69.4% and 72.0%, respectively.

### 2.3. Verification of the Genetic Transformation System Using Mature Wheat Embryos

#### 2.3.1. CRISPR/Cas9-Mediated Editing of the *Rht8* and *Rht12* Genes

**Figure 9:**
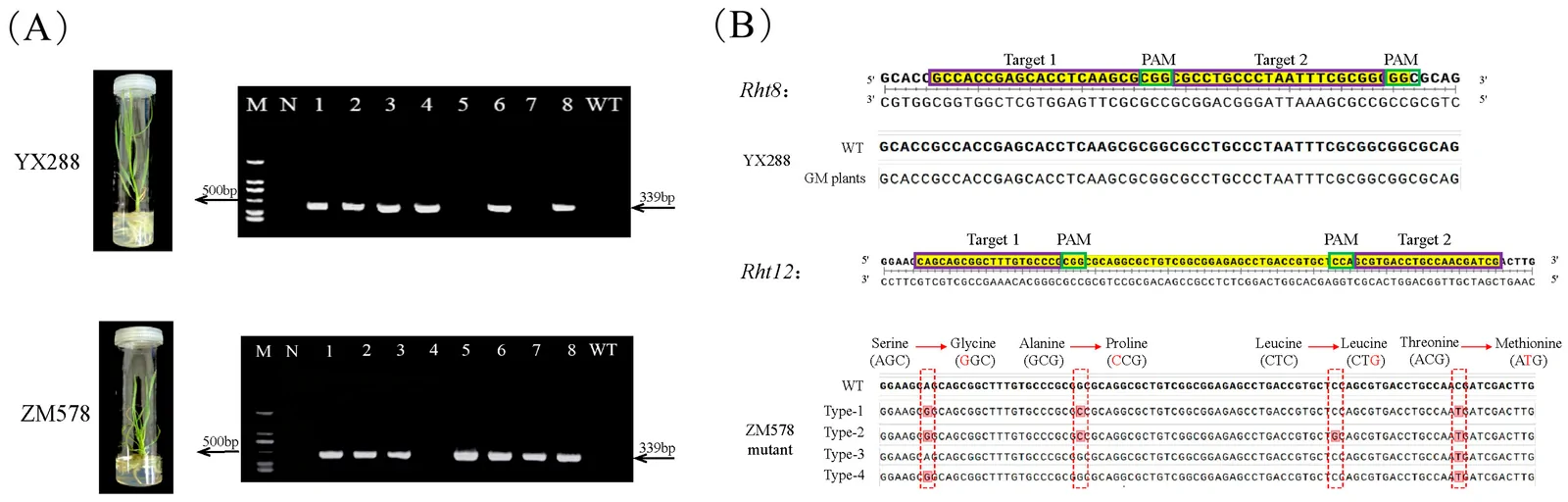
Sequencing verification of *Rht8* and *Rht12* gene editing in transgenic lines. (**A**): Transgenic test-tube seedlings and PCR detection, M is the DL2000 Marker, N is the negative control, lanes 1–8 represent transgenic seedling, and WT is the wild-type. (**B**): The upper panel shows the sequencing verification of the dual-target editing of *Rht8* in YX288. The lower panel displays the sequencing verification of the dual-target editing of *Rht12* in ZM578. GM plants indicate the transgenic lines. The red dashed boxes represent the mutated nucleotide sites. The purple and green highlighted regions represent the sgRNA target sequences and PAM sequences, respectively.

Using the stable transformation system based on mature embryos, we targeted the dwarfing genes *Rht8* in YX288 and *Rht12* in ZM578 by CRISPR/Cas9 editing (Figure 9A). In the T_0_ generation, PCR analysis identified 11 positive YX288 plants, corresponding to a positive rate of 61.11% and a transformation rate of 3.50%. In ZM578, 27 positive plants were identified, corresponding to a positive rate of 72.97% and a transformation rate of 2.25%. To verify editing at the target sites, the target regions of *rht8* and *rht12* were sequenced in all PCR-positive plants (Figure 9B). Four *rht12* mutation types were detected in ZM578, yielding a gene-editing efficiency of 37.8%. All detected mutations comprised simultaneous single-base substitutions at both target sites and resulted in multiple amino acid changes. These molecular analyses indicated that *Rht12* was successfully edited in ZM578 using the mature-embryo transformation system. In contrast, sequencing of all PCR-positive YX288 plants detected no substitutions, insertions, or deletions in either *rht8* target region, indicating that target-gene editing was unsuccessful.

#### 2.3.2. Comparison of Phenotypic Traits Between *rht8* and *rht12* Wheat Mutants

**Figure 10:**
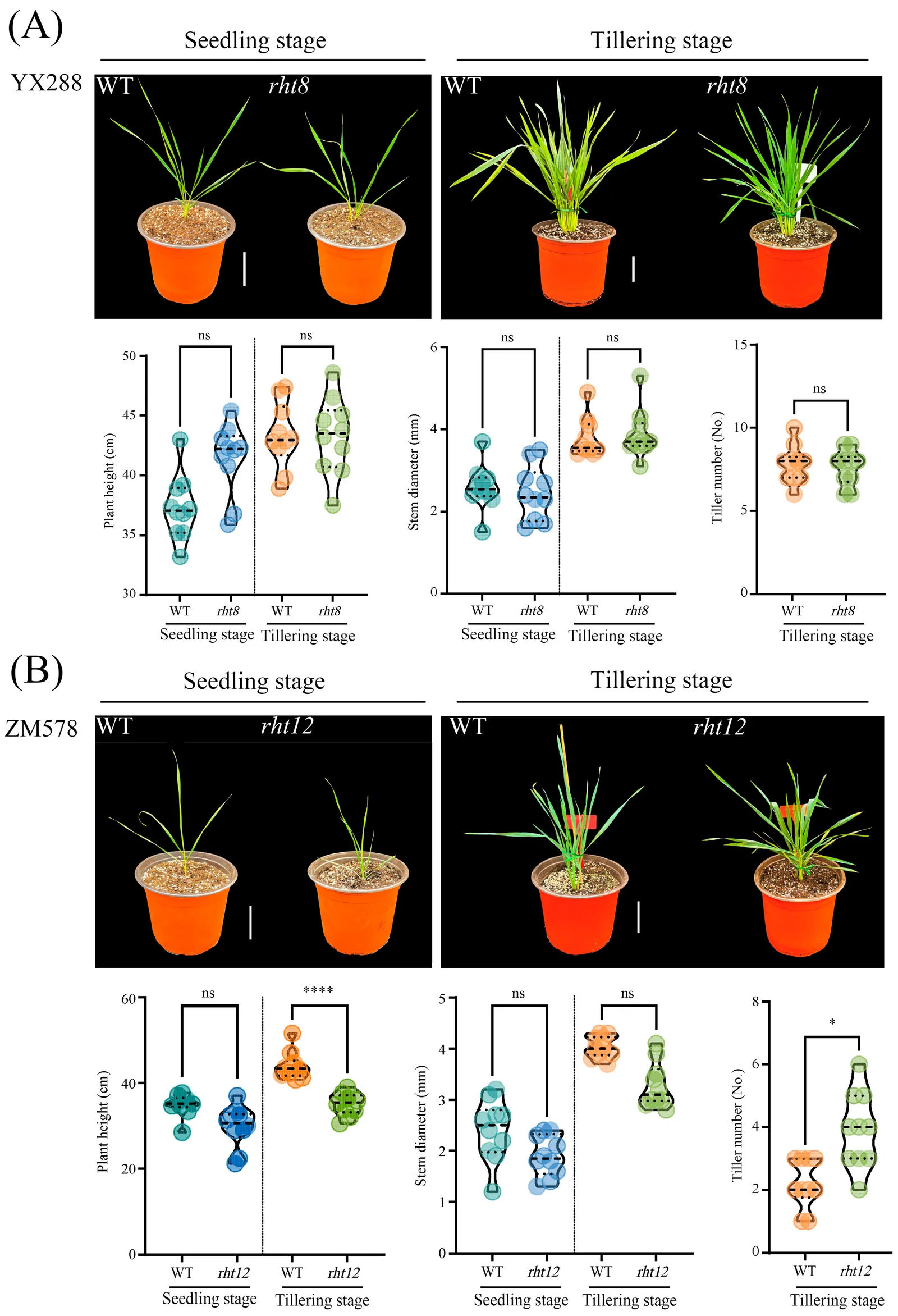
Phenotypic characteristics of transgenic lines. (**A**,**B**): Plant height, stem diameter and tiller number in the variety YX288 and ZM578, respectively, after target genes editing. The horizontal lines in the diagram represent the median of the data. ‘ns’ means no significant difference, * and **** represent *p* < 0.05 and *p* < 0.0001, respectively (*n* = 15).

Plant height, stem diameter, and tiller number were measured to characterize phenotypic variation in the *rht8* and *rht12* wheat mutants (Figure 10). At both the seedling and tillering stages, the growth traits did not differ significantly between WT and *rht8*-transformed YX288 plants, consistent with the absence of detectable *rht8* editing. In ZM578, plant height at the seedling stage and the stem diameter at both stages did not differ significantly between WT and *rht12*-mutant plants. However, at the tillering stage, *rht12* mutants were significantly shorter and produced significantly more tillers than WT plants. These phenotypic results were consistent with the molecular detection of four *rht12* mutant types.

## 3. Discussion

### 3.1. Regulatory Factors Affecting in Vitro Induction from Mature Wheat Embryos

Genotype dependence is a major bottleneck limiting in vitro culture and genetic transformation in bread wheat [2]. Previous studies have suggested that wheat genotypes from different ecological types may have different thresholds of sensitivity to auxin [13]. The winter variety Cadenza has a strict vernalization requirement and responded well to the lower dicamba concentration in A5. This response may reflect greater sensitivity to exogenous auxin, although endogenous auxin status was not measured in this study. In contrast, the semi-winter varieties ZM578 and YX288 responded best to the intermediate hormone combination in A7. The spring varieties Fielder and Bobwhite grow and develop rapidly and have high endogenous metabolic activity. Their optimal response to the higher dicamba concentration in A8 may reflect stronger auxin-inactivation or signal-attenuation mechanisms, although this interpretation requires experimental verification.

During differentiation, WF3 (1/2 MS) produced higher bud-differentiation rates across wheat varieties than WF2 (MS). One possible explanation is that the higher ionic strength of full-strength MS medium imposes greater osmotic stress on embryogenic cells, potentially interfering with cell polarity and fate reprogramming and promoting reactive oxygen species accumulation and callus browning [14,15]. The 1/2 MS medium may alleviate ionic stress while maintaining an adequate nutrient supply, thereby providing lower-osmotic-stress conditions favorable for cell division and green-bud differentiation [16].

During rooting culture, IBA markedly stimulated root-primordium differentiation and development, yielding regenerated plantlets with abundant fibrous roots and a large total root surface area [17]. In contrast, NAA primarily induced sparse, thick taproots and weakly stimulated lateral-root growth; it also promoted extensive lignification and deeper penetration of the primary roots [18]. Therefore, under adverse conditions such as drought, soil compaction, and high osmotic stress, NAA supplementation may strengthen root anchorage and improve plantlet stress tolerance and survival. Accordingly, IBA alone or a mixture of IBA and NAA may be selected to tailor root-system architecture [16], enabling the targeted optimization of regenerated plantlets.

Furthermore, following the *Agrobacterium*-mediated transformation of six wheat varieties, the semi-winter varieties YX288 and ZM578 exhibited high callus vitality and positive *GUS* expression. These findings indicated that their calli retained comparatively high physiological activity under transformation stress.

### 3.2. Agrobacterium-Mediated Transformation Efficiency Depends on the Coordinated Regulation of Multiple Factors

Low efficiency in *Agrobacterium*-mediated wheat transformation arises from two principal constraints. The first is the combined oxidative, mechanical, and metabolic stress induced by exogenous *Agrobacterium* infection. The second is the balance between cellular repair capacity and competence for plant regeneration [11,12].

Supplementing the coculture medium with AgNO_3_ significantly reduced callus browning and improved post-transformation survival and physiological activity. Ag+ inhibits ethylene action and may therefore attenuate infection-induced ethylene signaling, cellular senescence, and browning, allowing additional time for T-DNA integration and the repair of cellular damage [19,20]. Treatment T-2 (ultrasound + vacuum infiltration) provided moderate physical assistance and significantly increased the frequency and effective surface area of contact between *Agrobacterium* and plant cells without causing irreversible mechanical damage. Therefore, this treatment promoted efficient T-DNA transfer, consistent with previous reports on overcoming host-cell barriers in plants [21,22]. In contrast, excessive physical treatment (T-9) exacerbated the mechanical damage to cells and significantly increased contamination.

Tween20 can promote the uniform distribution of *Agrobacterium* over the callus surface by reducing the surface tension of the bacterial suspension. It can also facilitate the removal of excess suspension after infection, thereby reducing subsequent bacterial contamination [23]. PEG_6000_ may increase membrane permeability, facilitate T-DNA transfer, and provide osmotic protection against infection-related stress [16,19]. In this study, the combined addition of Tween20 and PEG_6000_ to the infection solution (D-3) produced the best callus recovery, suggesting that these additives jointly reduced physiological damage. The *Agrobacterium* suspension also had a clear optimal OD_600_ range of 0.6–0.8. At OD_600_ = 0.4, the efficiency of T-DNA delivery was low. At OD_600_ = 1.0, the calli produced numerous adventitious roots and showed inhibited green-bud regeneration. This response may reflect the excessive production of plant hormones or biostimulants at high *Agrobacterium* concentrations, which could disrupt the cytokinin-to-auxin balance and alter cell-fate specification [6,14].

Despite these optimization strategies, the overall rate of transient *GUS* expression remained relatively low. This recalcitrance may largely reflect the developmental state of mature embryos, which contain highly differentiated cells with limited competence for *Agrobacterium* infection and T-DNA integration. Wheat cells also typically mount strong basal defense responses—including marked reactive oxygen species (ROS) bursts and programmed cell death—upon recognition of *Agrobacterium*, thereby limiting transgene expression [11,12]. To overcome the remaining bottleneck in mature-embryo transformation, future studies could use morphogenic regulators to reprogram cell fate and stimulate meristematic activity [1,2,5]. The transient co-expression of immune-suppressing or ROS-scavenging effectors during early infection may also mitigate host defenses and improve T-DNA delivery and stable integration.

### 3.3. Validation of the Transformation System and Phenotypic Analysis of Gene-Edited Mutants

The optimized protocol circumvented the seasonal limitations associated with immature-embryo collection and produced transformation efficiencies of 1.8–2.5% in the two semi-winter varieties. These values are promising relative to the 0.33–2.33% reported for cultivar Amal [24]. However, because the protocol was validated in only a limited number of genotypes, additional wheat varieties should be tested to determine its broader applicability. To evaluate the utility of the platform, two elite semi-winter varieties were used for targeted gene editing. CRISPR/Cas9-mediated targeting of *Rht12* in ZM578 produced multiple single-base substitutions. The *rht12* mutants significantly reduced plant height and increased tiller number at the tillering stage, resulting in compact, dwarf, and high-tillering phenotypes. The stable editing outcome enabled the phenotypic characterization of the recovered plants. In contrast, after positive testing, the transgenic plants of the *Rht8* gene in variety YX288 were successfully obtained. *Rht8* editing was unsuccessful in YX288, possibly because of genotype-specific effects or insufficient cleavage efficiency of the selected sgRNA. Future studies should use multiple sgRNAs or multiplex-editing strategies and evaluate the *Rht8* target in additional genotypes.

## 4. Materials and Methods

### 4.1. Plant Materials and Experimental Design

This study used 8 major wheat varieties cultivated in the Huang-Huai-Hai region (Table S1): the winter varieties Cadenza and LM14, the semi-winter varieties FM5, XM26, ZM578, and YX288, and the spring varieties Fielder and Bobwhite. The varieties were planted at the experimental station of Anhui Science and Technology University in October 2023 using a sequential field design. Standard irrigation, fertilization, and pest and disease management practices were applied to support plant growth and grain filling. The varieties were harvested in June 2024. After harvest, plump seeds free of pests, disease symptoms, and mechanical damage were air-dried to a moisture content of 12–13% and stored at 4 °C until mature embryos were excised as explants.

### 4.2. Construction of Regeneration Systems for Winter, Semi-Winter, and Spring Wheat

#### 4.2.1. Callus Induction and Differentiation Culture for the Three Wheat Types

Mature seeds from the 8 varieties were selected, placed in sterile 50 mL centrifuge tubes, and surface-sterilized sequentially. The seeds were shaken in 70% ethanol for 5 min; the ethanol was then discarded. The seeds were subsequently shaken in 0.1% HgCl_2_ solution for 15 min, washed 4–5 times with sterile water, and soaked overnight at 4 °C after the final wash. After soaking, the seeds were shaken in 10% NaClO solution for 5 min and then washed 4–5 times with sterile water. All shaking steps were performed at 180 rpm. Surface moisture was removed from the disinfected seeds using sterile filter paper.

Scutella were excised using a dissecting needle and inoculated onto induction medium. Each Petri dish (90 × 15 mm) was inoculated with 20–30 scutella. The dishes were sealed and cultured in darkness in a tissue-culture room for 14 days to induce callus formation; the callus induction rate was then calculated. Under aseptic conditions, embryogenic calli with vigorous growth, a dense structure, pale-yellow spherical morphology, and suitable firmness were selected and transferred to fresh induction medium for an additional 7 days of subculture in darkness. Callus fresh weight was measured, and the calli were dried at 63 °C for dry-weight determination. The moisture content and comprehensive score (V) were then calculated. The tissue-culture room was maintained at 26 ± 2 °C and 60–70% relative humidity under continuous darkness. Induction media contained different combinations of 2,4-D and dicamba, yielding 16 treatments (A1–A16; Table S2). All media were based on MS medium supplemented with 150.0 mg/L asparagine (Asn), 500.0 mg/L hydrolyzed casein (CH), 2.0 mg/L picloram, 30.0 g/L sucrose, and 8.0 g/L agar (pH = 5.8). The calli were transferred to differentiation medium and cultured under light for 30 days, after which the differentiation rate was determined. Three differentiation media—WF2, WF3, and WF4—were evaluated (Table 2).

#### 4.2.2. Rooting Culture of Regenerated Seedlings from the Three Wheat Types

After 30 days of callus differentiation, regenerated seedlings of the 8 varieties with uniform, vigorous growth were selected, inoculated into rooting medium, and cultured under light for an additional 30 days. The root morphological traits were quantified using a root scanner (Regent Instruments, Quebec, QC, Canada), including total root length, root surface area, root volume, average root diameter, number of root tips, and number of root bifurcations. Three rooting treatments, WR1, WR2, and WR3, were evaluated (Table 3).

### 4.3. Construction of the Wheat Transformation System

#### 4.3.1. Preparation of the Infection Solution

The overexpression vector pCAMBIA2301-*GUS* (CaMV35S promoter) and the CRISPR/Cas9 editing vector K42-CCDB-Δ*Rht12/Rht8* (OsU3 promoter) were provided by our laboratory. The plasmids were introduced into *Agrobacterium* strain *EHA105* (Sangon Biotech, Shanghai, China). Briefly, positive transformants were streaked onto solid LB medium containing 50 mg/L Kan^+^ and 100 mg/L Rif^+^ and were incubated at 28 °C in darkness for 2–3 days. A single colony was then inoculated into 50 mL of liquid LB medium containing the same antibiotics. After incubation at 28 °C with shaking at 200 rpm for 24 h, bacterial cells were collected by centrifugation, resuspended in infection buffer containing 200 µmol/L acetosyringone and 0.02% Silwet L-77, and adjusted to OD_600_ = 0.5 for subsequent use [6]. The formulations of the solid LB medium, liquid LB medium, and infection buffer are provided in Table S3.

#### 4.3.2. Infection and Recovery Culture of Calli

Based on the optimized regeneration system described above, the calli of the model variety Cadenza were collected and infected with *Agrobacterium* by shaking for 3 h (28 °C, 180 rpm). The explants were then transferred to coculture medium supplemented with 4 mg/L AgNO_3_; medium without AgNO_3_ served as the control. After coculture for 3 days (28 °C, darkness), the calli were washed 2–3 times with sterile water, blotted on sterile filter paper to remove surface moisture, and transferred to recovery medium for 15 days in darkness; the medium was renewed every 7 days. The calli were subjected to transient *GUS* staining to calculate the *GUS* staining rate and were then transferred to differentiation medium for further culture.

In parallel, the calli of Cadenza that had been induced for 21 days were immersed in infection solution containing pCAMBIA2301-*GUS* and subjected to different combinations of ultrasound and vacuum infiltration (9 treatments, T-1 to T-9; Table S4) to improve infection efficiency. The calli were then infected by shaking for an additional 3 h (28 °C, 180 rpm) and transferred to coculture medium containing 4 mg/L AgNO_3_ for 3 days (28 °C, darkness). After being washed 2–3 times with sterile water and blotted on sterile filter paper, the calli were transferred to recovery medium for 15 days in darkness; the medium was renewed every 7 days. Callus contamination and transient *GUS* staining rates were determined. The calli were then transferred to differentiation medium for further culture. The compositions of the initial induction, subculture induction, coculture, recovery, and differentiation media are provided in Table S3.

Following parameter optimization, *Agrobacterium* carrying pCAMBIA2301-*GUS* was used to infect the six varieties described above. Infection was assisted by ultrasound and vacuum infiltration (T-2: ultrasound for 2.5 min and vacuum infiltration for 5 min). The calli were then infected by shaking for an additional 3 h and transferred to coculture medium containing 4 mg/L AgNO_3_ for 3 days (28 °C, darkness). After the calli had been washed 2–3 times with sterile water and blotted on sterile filter paper, their initial fresh weight (W_i_) was measured. The calli were then cultured on recovery medium for 15 days in darkness; the medium was renewed every 7 days. Fresh weight (Wr), callus vitality, and the *transient GUS* staining rate were determined. After 40 days on differentiation medium, the two superior varieties, YX288 and ZM578, were selected for further optimization.

### 4.4. Optimization of Infection Parameters for YX288 and ZM578

#### 4.4.1. Optimization of Additives in the Infection Solution

Based on the reported infection-suspension concentration of OD600 = 0.5, the following additives were evaluated: 0.02% Silwet L-77 (D-1), 0.02% Silwet L-77 + 250 mg/L PEG_6000_ (D-2), and 0.01% Tween20 + 250 mg/L PEG_6000_ (D-3) (Table S5). In combination with ultrasound and vacuum infiltration (T-2), the explants were shaken for 3 h and then transferred to coculture medium containing 4 mg/L AgNO_3_ for 3 days (28 °C, darkness), during which callus condition was monitored. After the calli were washed 2–3 times with sterile water and blotted on sterile filter paper, their initial fresh weight (Wi) was measured. After 15 days of recovery culture in darkness, with medium renewal every 7 days, callus fresh weight (Wr) and dry weight (Dr) were measured to calculate moisture content and callus vitality.

#### 4.4.2. Optimization of the Infection-Suspension Concentration

Four concentrations of the infection suspension were evaluated: OD600 = 0.4, 0.6, 0.8, and 1.0. Infection was assisted by ultrasound and vacuum infiltration (T-2). The calli were shaken for 3 h and transferred to coculture medium containing 4 mg/L AgNO_3_ for 3 days (28 °C, darkness). They were then washed 2–3 times with sterile water, blotted on sterile filter paper, and cultured on recovery medium for 15 days in darkness; the medium was renewed every 7 days. After recovery, light-yellow, compact, contamination-free calli were selected. Fresh weight (Wr) was measured, and calli were stained with *GUS* solution to determine the staining rate. The calli were then transferred to differentiation medium for 40 days to promote clustered-bud growth. Differentiation and shoot regeneration rates were recorded.

### 4.5. Identification of Transgenic Plants

#### 4.5.1. *GUS* Staining

*GUS* staining solution was prepared according to the manufacturer’s instructions (PH1727, PHYGENE, Fujian, China) [25]. Calli were fixed in 90% acetone, washed twice with *GUS* staining buffer, immersed in *GUS* staining solution (X-Gluc), and vacuum-infiltrated for 30 min. The calli were then incubated in the staining solution for 12 h in darkness at 37 °C, decolorized 2–3 times with 75% anhydrous ethanol while shaking at 180 rpm, and observed and photographed under a microscope.

After 100 days of transgenic seedling growth, young leaves and root tips were stained with *GUS* solution and decolorized. The samples were placed on glass slides and gently compressed to prepare specimens, which were examined under a dissecting microscope (YSZ710, Stereomicroscope, Hefei, Anhui, China).

#### 4.5.2. PCR Detection and Mutation Site Identification

After transformation with the pCAMBIA2301-*GUS* overexpression vector or the K42-CCDB-Δ*Rht12*/*Rht8* CRISPR/Cas9 editing vector and 120 days of growth, the transgenic wheat plants were labeled individually. Leaves from T0-generation plants were collected, immediately frozen in liquid nitrogen, and ground to a powder. Genomic DNA was extracted using the CTAB method. The *GUS*-PTF1/R1 primers were used to detect the *GUS* gene, and the Cas9-339F/R primers were used to detect the CRISPR/Cas9 vector; detailed primer information is provided in Table S6. The PCR reaction mixture and amplification program followed the procedure described by Dai [25]. The PCR-positive rate was calculated. Target regions were amplified from PCR-positive plants using the *rht8*-Ta322F/R and *rht12*-Ta254F/R primers, and the amplicons were sequenced to identify mutations.

#### 4.5.3. Evaluation of Agronomic Traits in *rht12*/*rht8* Mutant Plants

T_0_-generation mutant and wild-type (WT) plants were grown under greenhouse conditions. Major vegetative growth-related traits, including plant height, stem diameter, and tiller number, were measured at the seedling and tillering stages.

### 4.6. Data Analysis

#### 4.6.1. Analysis of Callus Induction and Transformation

The indicators used to evaluate callus induction, differentiation, regeneration, and genetic transformation were calculated by using the following formulas (Table 4):

#### 4.6.2. Comprehensive Evaluation of the Callus Induction Effect

SPSS 18.0 software was used to standardize the callus fresh weight, dry weight and induction rate and to calculate the comprehensive score (V). These values were used to evaluate the callus-induction capacity of different treatments and wheat varieties and to identify the optimal treatment and genotype [2,25,26].

(1) Standardized score (M) = (X_i_ − X_min_)/(X_max_ − X_min_)

Note: X_i_ represents the observed value of a given indicator, and X_min_ and X_max_ represent the minimum and maximum observed values, respectively.

(2) Comprehensive score (V) = (Induction rate score × 30%) + (Dry weight score × 50%) + (Fresh weight score × Dynamic weight) [27]

Note: The dynamic weight was adjusted according to callus moisture content. If moisture content exceeded 90%, a weight of 10% was assigned; otherwise, a weight of 20% was assigned.

Differences among treatments or varieties were analyzed using SPSS 18.0. Duncan’s multiple range test was used for multiple comparisons, and Student’s t test was used to compare WT and mutant plants in Figure 10. Graphs were prepared using GraphPad Prism 8.

## 5. Conclusions

Using mature wheat embryos as explants, this study established tissue-culture systems tailored to three ecological types by screening eight wheat varieties. Treatments A5, A7, and A8 were the most suitable callus-induction media for winter, semi-winter, and spring varieties, respectively. A differentiation medium based on 1/2 MS was broadly applicable across ecological types, and IBA was the most effective treatment for rooting regenerated seedlings. The optimal transformation conditions combined ultrasound (2.5 min) and vacuum infiltration (5 min), supplementation of the infection solution with PEG_6000_ (250 mg/L) and Tween20 (0.01%), and an OD_600_ of 0.6–0.8. Across multiple varieties, transformation efficiencies were 2.5% for YX288 and 1.8% for ZM578. Using this transformation system, *Rht12* in ZM578 and *Rht8* in YX288 were targeted by CRISPR/Cas9. ZM578 *rht12* mutants carrying single-base substitutions at multiple target sites exhibited significantly reduced height, increased tillering, and a compact plant architecture, whereas no successfully edited *rht8* mutants were obtained in YX288. These findings indicate that a reliable mature-embryo transformation system was established for wheat and that gene editing was successfully validated in the semi-winter variety ZM578. This system provides technical support for further functional analyses of key genes in bread wheat.

## Figures and Tables

**Table 1:** Root-related indicators of regenerated seedlings of eight wheat varieties under different rooting media.

Variety	Total Root Length (cm)			Root Surface Area (cm^2^)			Root Volume (cm^3^)			Avg Root Diameter (mm)			Root Tips (No.)			Root Branches (No.)		
	NAA	IBA + NAA	IBA	NAA	IBA + NAA	IBA	NAA	IBA + NAA	IBA	NAA	IBA + NAA	IBA	NAA	IBA + NAA	IBA	NAA	IBA + NAA	IBA
Cadenza	16.456 ± 1.764 c	31.046 ± 4.459 b	63.766 ± 4.135 a	3.321 ± 0.258 c	4.024 ± 0.330 b	7.179 ± 0.349 a	0.056 ± 0.007 b	0.040 ± 0.004 c	0.073 ± 0.007 a	0.711 ± 0.062 a	0.450 ± 0.039 b	0.393 ± 0.018 c	28.600 ± 2.596 c	64.100 ± 8.078 b	153.100 ± 12.655 a	84.900 ± 5.128 b	78.600 ± 6.015 b	322.100 ± 23.075 a
LM14	20.867 ± 2.344 c	35.518 ± 4.321 b	66.851 ± 4.873 a	3.677 ± 0.252 b	3.992 ± 0.317 b	7.202 ± 0.400 a	0.054 ± 0.005 b	0.041 ± 0.004 c	0.079 ± 0.008 a	0.717 ± 0.048 a	0.543 ± 0.042 b	0.397 ± 0.022 c	33.900 ± 2.759 c	70.600 ± 6.028 b	158.300 ± 11.666 a	91.800 ± 5.517 b	85.300 ± 5.679 b	332.800 ± 20.940 a
FM5	26.024 ± 2.329 c	33.627 ± 4.537 b	65.579 ± 4.449 a	3.523 ± 0.283 c	4.028 ± 0.286 b	7.088 ± 0.326 a	0.055 ± 0.005 b	0.042 ± 0.004 c	0.081 ± 0.007 a	0.729 ± 0.045 a	0.519 ± 0.044 b	0.402 ± 0.018 c	34.100 ± 2.554 c	67.000 ± 6.011 b	159.000 ± 10.127 a	93.500 ± 4.121 b	76.800 ± 5.701 c	327.700 ± 22.292 a
XM26	24.202 ± 3.224 c	34.595 ± 3.815 b	63.168 ± 4.850 a	3.388 ± 0.281 c	3.968 ± 0.307 b	7.084 ± 0.397 a	0.053 ± 0.006 b	0.043 ± 0.004 c	0.081 ± 0.008 a	0.721 ± 0.049 a	0.537 ± 0.041 b	0.412 ± 0.019 c	35.300 ± 3.099 c	70.500 ± 5.764 b	155.400 ± 12.033 a	95.200 ± 5.341 b	84.100 ± 5.634 c	337.000 ± 19.702 a
YX288	24.712 ± 2.972 c	34.245 ± 4.643 b	64.398 ± 4.070 a	3.601 ± 0.251 c	4.022 ± 0.284 b	7.084 ± 0.335 a	0.058 ± 0.004 b	0.041 ± 0.004 c	0.081 ± 0.009 a	0.717 ± 0.046 a	0.503 ± 0.047 b	0.408 ± 0.017 c	34.600 ± 2.911 c	67.500 ± 5.911 b	159.600 ± 9.095 a	98.400 ± 5.231 b	79.400 ± 5.726 c	336.300 ± 21.161 a
ZM578	22.980 ± 2.906 c	32.897 ± 4.113 b	64.431 ± 4.771 a	3.545 ± 0.243 c	3.990 ± 0.320 b	7.051 ± 0.340 a	0.054 ± 0.005 b	0.043 ± 0.004 c	0.081 ± 0.007 a	0.719 ± 0.044 a	0.537 ± 0.047 b	0.413 ± 0.018 c	34.800 ± 2.796 c	71.100 ± 5.857 b	158.800 ± 11.394 a	92.600 ± 4.334 b	81.600 ± 5.508 c	340.800 ± 19.234 a
Fielder	20.613 ± 2.912 c	33.301 ± 3.826 b	66.205 ± 4.315 a	3.455 ± 0.302 c	3.996 ± 0.263 b	7.016 ± 0.282 a	0.055 ± 0.005 b	0.041 ± 0.004 c	0.082 ± 0.008 a	0.715 ± 0.044 a	0.526 ± 0.046 b	0.412 ± 0.016 c	35.100 ± 2.714 c	67.200 ± 6.168 b	159.100 ± 9.570 a	97.400 ± 5.016 b	79.100 ± 5.231 c	335.800 ± 20.644 a
Bobwhite	22.191 ± 2.965 c	36.901 ± 3.777 b	66.337 ± 4.651 a	3.545 ± 0.259 c	3.998 ± 0.308 b	7.085 ± 0.366 a	0.054 ± 0.005 b	0.041 ± 0.004 c	0.081 ± 0.009 a	0.733 ± 0.049 a	0.530 ± 0.048 b	0.411 ± 0.016 c	34.400 ± 2.717 c	71.400 ± 5.518 b	160.200 ± 11.158 a	92.400 ± 5.298 b	82.500 ± 5.464 c	330.600 ± 21.308 a

Note: Data are presented as mean ± standard error (*n* = 10). For each wheat variety, lowercase letters represent the significant differences among the three rooting media, according to Duncan’s multiple range test (*p* < 0.05).

**Table 2:** Differentiation treatments.

Treatments	Medium Components
WF2	MS medium+ 0.3 g/L CH + 1.5 mg/L KT + 0.2 mg/L NAA + 30 g/L Sucrose + 8.0 g/L Agar (pH = 5.8)
WF3	1/2 MS medium + 0.3 g/L CH + 1.5 mg/L KT + 0.2 mg/L NAA + 30 g/L Sucrose + 8.0 g/L Agar (pH = 5.8)
WF4	1/4 MS medium + 0.3 g/L CH + 1.5 mg/L KT + 0.2 mg/L NAA + 30 g/L Sucrose + 8.0 g/L Agar (pH = 5.8)

Note: The plant growth regulators and additives are abbreviated as follows: KT, kinetin; NAA, 1-naphthaleneacetic acid; CH, hydrolyzed casein.

**Table 3:** Rooting treatments.

Treatments	Basal Components of Culture Media	Hormones (mg/L)	
		IBA	NAA
WR1	1/2 MS medium + 20.0 g/L Sucrose + 4.0 g/L Plant gel (pH = 5.8).	0.2	
WR2			0.2
WR3		0.2	0.2

Note: IBA, indole-3-butyric acid; NAA, 1-naphthaleneacetic acid.

**Table 4:** Calculation of relevant indicators.

Number	Index	Formula
(1)	Callus tissue vitality (%)	(W_r_-W_i_)/W_i_ × 100
(2)	Moisture content (%)	(1-(D_r_/W_r_)) × 100
(3)	*GUS* staining rate (%)	(Calli number with blue spots/total number of detected calli) × 100
(4)	Differentiation rate (%)	(Differentiated calli number/Total calli number) × 100
(5)	Shoot regeneration rate (%)	(Bud calli number/Total calli number) × 100
(6)	Contamination rate (%)	(Contamination calli number/Total calli number) × 100
(7)	Induction rate (%)	(Total calli number/Total explant number initially inoculated) × 100
(8)	Seedling regeneration rate (%)	(Regenerated plant number with surviving after transplanting to soil/Total explant number initially inoculated) × 100
(9)	Plant transformation rate (%)	(Positive plants number detected by PCR/Total explant number initially inoculated) × 100
(10)	Positive rate (%)	(Positive plants number detected by PCR/Plants number of T_0_ generation) × 100

Note: W_i_ is the initial fresh weight of the calli after *Agrobacterium* coculture, W_r_ is the fresh weight after recovery culture, and D_r_ is the dry weight after recovery culture.

## Data Availability

The data that support the findings of this study are available from the corresponding author upon reasonable request.

## Supplementary Materials

The following supporting information can be downloaded at: https://www.mdpi.com/article/10.3390/plants15182766/s1, Table S1: Varieties origin of bread wheat, Table S2: Induction treatments of 2,4-D and Dicamba, Table S3: Medium formula of transformation system, Table S4: Auxiliary infection treatment of ultrasonication and vacuum infiltration, Table S5: Supplementation of different additives in Agrobacterium infection solution, Table S6: Primers used for gene editing.
